# Chloroquine Improves Deoxynivalenol-Induced Inflammatory Response and Intestinal Mucosal Damage in Piglets

**DOI:** 10.1155/2020/9834813

**Published:** 2020-06-09

**Authors:** Simeng Liao, Shengguo Tang, Bie Tan, Jianjun Li, Ming Qi, Zhijuan Cui, Andong Zha, Yanan Wang, Yulong Yin, Peng Sun, Yulong Tang

**Affiliations:** ^1^Laboratory of Animal Nutritional Physiology and Metabolic Process, Key Laboratory of Agro-ecological Processes in Subtropical Region, National Engineering Laboratory for Pollution Control and Waste Utilization in Livestock and Poultry Production, Institute of Subtropical Agriculture, Chinese Academy of Sciences, Changsha, 410125 Hunan, China; ^2^State Key Laboratory of Animal Nutrition, Institute of Animal Science, Chinese Academy of Agricultural Sciences, Beijing 100193, China; ^3^University of Chinese Academy of Sciences, Beijing 100008, China; ^4^College of Animal Science and Technology, Hunan Agricultural University, Changsha 410128, China; ^5^College of Animal Nutrition and Feed Science, Huazhong Agricultural University, Wuhan, 430070 Hubei, China

## Abstract

We investigated the effects of rapamycin (RAPA) and chloroquine (CQ) in supporting growth performance and the intestinal mucosal barrier in response to deoxynivalenol (DON) in piglets. A total of 32 healthy weaned piglets (bodyweight 7.10 ± 0.58 kg) were divided into four groups and treated daily with RAPA (1 mg/kg BW), CQ (10 mg/kg BW), or a control volume of normal saline (two groups) until the end of the experiment. After feeding a basal diet for seven days, three groups were then switched to mildewed feed containing 1 mg kg/DON for a further seven days. In contrast to the control group, DON-treated piglets showed decreased average daily gain (ADG) and daily feed intake (ADFI), as well as negatively affected intestinal morphology as indicated by villus height, crypt depth, and tight junction protein expression. A group treated with RAPA and DON showed increased intestinal autophagy, aggravated inflammatory responses, and damage to the intestinal mucosa and permeability, leading to reduced growth performance. Meanwhile, a group treated with CQ and DON showed indices comparable to the non-DON control group, with alleviated inflammatory cytokines and healthy intestinal morphology and structure. They also showed better growth performance compared to DON treatment alone. These findings have important implications for mediating autophagy against DON *in vivo*, as well as the potential for CQ in improving growth performance and maintaining intestinal barrier integrity in weanling piglets.

## 1. Introduction

Vomitoxin, known as deoxynivalenol (DON) [1], is one of the several mycotoxins produced by various *Fusarium* species that frequently grow on corn, wheat, oats, barley, rice, and other grains in the field or during storage. It is widely found in food and animal feed, which is harmful to human health and animal husbandry development. DON shows cytotoxic, genotoxic, and visible antiproliferative effects in intestinal cells, affecting cell cycle distribution, inducing apoptosis, and inhibiting the synthesis of biomacromolecules [2–4]. These effects occur after the ingestion of contaminated foods from affected grains, such as acute temporary nausea, vomiting, diarrhea, abdominal pain, headache, dizziness, and fever, with the gastrointestinal tract as the primary target in reared animals [5–8]. Moreover, DON inhibits the absorption of nutrients and damages intestinal epithelial cells and eventually affects material deposition [9–11].

Autophagy is a catabolic process aimed at recycling cellular components and damaged organelles in response to diverse conditions of stress, such as nutrient deprivation, viral infection, and genotoxic stress. It is upregulated by cellular stressors to enhance cell survival [12, 13]. We previously found that autophagy plays a crucial role in maintaining intestinal epithelial cell homeostasis under DON stress, and DON induces autophagy in pig intestinal cells (IPEC-J2). The knockout of the ATG5 gene led to autophagy deficiency and increased apoptosis via the generation of reactive oxygen species, suggesting that intestinal cell autophagy is essential in protecting against DON [14, 15].

It is of interest to understand the role of autophagy to intestinal barrier function, immunity, and inflammation following the exposure to DON *in vivo*. We attempted to intervene in autophagy using the autophagy activator rapamycin (RAPA) and inhibitor chloroquine (CQ) to understand the relationship between autophagy and mycotoxins in piglets. RAPA, a macrolide antibiotic, is a widely used inhibitor of the mammalian target of rapamycin (mTOR), which induces autophagy in a variety of cell types [16], while CQ is a late-stage inhibitor of autophagy, which inhibits autophagic flux by blocking the fusion of autophagosomes and lysosomes [17]. The two reagents, RAPA and CQ, have often been used to mediate autophagy in the animal. In this report, after testing different dose, we chose the optimal concentration (1 mg/kg for RAPA and 10 mg/kg for CQ) [18, 19], which succeed in mediating intestinal autophagy level and showed different growth performance, to examine the effect of modulation of autophagy as a strategy to protect in mycotoxin stress. These findings provided evidence of the roles of RAPA and CQ in DON-exposed piglets and indicated a link between autophagy and alteration of the inflammatory response as well as intestinal morphology *in vivo*.

Through this experiment, DON induced intestinal cell autophagy, but excessive autophagy due to the addition of RAPA did not provide protection and instead aggravated intestinal disorders and induced higher levels of proinflammatory cytokines in both blood and intestinal tissue. Meanwhile, CQ downregulated autophagy and alleviated toxin damage and inflammatory condition relative to the control group.

## 2. Materials and Methods

### 2.1. Materials and Reactions

The *F. graminearum* R6576 strain, from Huazhong Agricultural University molecular biotechnology laboratory preserved in PDA cant. The RAPA and CQ components (purity 94 ≥ 98%) obtained from Sangon Biotech Co., Ltd. (Shanghai, China).

### 2.2. Preparation for Mildew Feed

Firstly, the mycelia were activated at 25°C ~ 28°C for 7 days in the dark. Secondly, then mycelia were inoculated in CMC liquid medium and cultured in shaker under continuous light condition (28°C, 200r). After 5 days, when conidium produced, the mycelia were filtered with aseptic double layer gauze and count the conidium with a blood count plate. Then, the concentration of conidia was adjusted to 5^∗^10^5^/mL and inoculated in the feed for 14 days. Finally, deoxynivalenol (DON) and zearalenone content in the fermented feed was detected by ELISA, and the proportion of zearalenone only contained 4.15%. Thus, we only count DON and diluted to the target concentration of 1 mg/kg with the basic diet.

### 2.3. Animals and Experimental Design

A total of 32 Large White × Landrace piglets weaned at 24 days (d) of age (average initial body weight (BW) of 7.10 ± 0.58 kg) were divided into four groups. Each group has 6 replicates with 1 pig per replicate, and there was no significant difference in average BW among three treatments. Treatments were as follows: RAPA (1 mg/kg BW) + DON, CQ (10 mg/kg BW) + DON, normal saline + DON, and control of normal saline. Adaptation to experimental diets took place from days 1 to 3. Then, piglets were orally administrated with 7 mL saline, RAPA or CQ from days 4 to 10 and took a basal diet. On day 11, the total volume of solution was adjusted according to the average weight of each treatment, and mildewed feed containing 1 mg kg/DON took place from days 11 to 17. Every single pig was housed in a cage equipped with a feeder for ingestion and a nipple drinker for free access to drinking water. The basal diet was formulated to meet the nutrient requirements for weanling piglets (NRC, 2012) and has been described in a previous study [20].

### 2.4. Sample Collection and Preparation

On day 14, final body weight and average daily feed intake (ADFI) were collected after the initial treatment, otherwise, calculating the ADG and gain to feed ratio (G/F). Ten milliliters of blood was collected from the jugular vein. Blood without anticoagulant was allowed to clot for 1 h at room temperature, and then serum was separated and stored as previously described [21], while blood with anticoagulant was for collecting plasma. After piglets were euthanized, jejunal and ileal samples (2 cm, jejunum as the 1/3 mid and ileum as 1/3 distal part) were collected for the determination of intestinal morphology. Then, samples for histological slicing were rapidly fixed with 10% neutral buffered formalin. An approximate 0.5 cm sample of the jejunum and ileum were immediately and rapidly excised with ice-cold physiological saline [22], then stored in the form of formaldehyde solution or at 2.5% glutaraldehyde solution until further analysis.

### 2.5. Western Blotting Analysis

Relative protein levels for Sequestosome 1 (P62/SQSTM1, P62), LC3, and *β*-actin in the jejunum were determined followed the methods of Simeng [23]. The primary antibodies used in the present study were as follows: anti-P62 (#23214), anti- LC3B, and anti-*β*-actin (#4970). Chemiluminescent reagent (BeyoECL Plus, Beyotime, Shanghai, China) with a ChemiDoc™ Touch Imaging System (Bio-Rad, Philadelphia, PA, USA) visualizing the bands of the protein. The resultant signals quantified as previously by [24]. Antibodies purchased from Cell Signaling Technology (Danvers, MA).

### 2.6. Analysis of Serum Concentrations of Cytokines, Intestinal Permeability Related Indicators

We followed the methods of Simeng Liao et al. [23] to determined serum concentrations of immunoglobulin (Ig) G, IgM (IgG and IgM quantitation kit; Bethyl Laboratories, Inc., Montgomery, TX, USA), tumor necrosis factor- (TNF-) *α*, interferon- (IFN-) *γ*, interleukin- (IL-) 1*β*, IL-6, IL-8, IL-10, IL-12 and transforming growth factor- (TGF-) *β* (Cell Biolabs, San Diego, CA, USA), and serum diamine oxidase (DAO) and D-lactate.

### 2.7. Intestinal Morphology

Intestinal morphology analyses were followed by the methods of [23]. Crypt depth, villous height, intestine wall thickness, goblet cell, and lymphocyte measured and counted with computer-assisted microscopy (Micro metrics TM; Nikon ECLIPSE E200, Tokyo, Japan).

### 2.8. Real-Time Quantitative Reverse Transcriptase PCR

Quantitative real-time PCR (qRT-PCR) was followed by the methods of [23]. In brief, total RNA was extracted using the Trizol reagent (Invitrogen, Thermo Fisher Scientific, USA). RNA was purified extracted using the RNeasy Mini Kit (Takara Bio Inc., Shiga, Japan), and remove contaminants with DNase (Takara, Shuzo, Kyoto, Japan). Then, the quality and quantity of RNA should be determined by ultraviolet spectroscopy (Nanodrop 2000 Spectrophotometer, Thermo Scientific, Courtaboeuf, France). The cDNA library was constructed using the TransScript One-Step gDNA Removal and cDNA Synthesis SuperMix kit (Transgen, China), then diluted as instructions. Amplification conditions were performed as follows: (1) denaturation for 10 min at 95°C; (2) 40 PCR cycles of denaturation for 15 s at 95°C, annealing for 15 s at 56–64°C and extension for 45 s at 56–64°C [23]. All PCR primers used in this study are listed in Table 1.

### 2.9. Immunohistochemical Analysis

The expressions of proliferating cell nuclear antigen (PCNA) and tight junction proteins as ZO-1, occludin, claudin-3, and claudin-1 were determined using immunohistochemical analysis as described by [25].

The stained sections were incubated with the primary antibodies described by [25]: anti-PCNA (1 : 200; Changsha Kainuo Biological Technology Co., Ltd., Changsha, China), ZO-1 polyclonal antibody (1 : 100; Abcam, Cambridge, UK), occludin polyclonal antibody (1 : 100; Abcam), claudin-3 poly-clonal antibody (1 : 100; Abcam), claudin-1 polyclonal antibody (1 : 100; Abcam), E-claudin poly-clonal antibody (1 : 100; Abcam), Rabbit hypersensitivity 2-step immunohistochemical kit (Boster Biological Technology), diaminobenzidine (Boster Biological Technology), and hematoxylin (Boster Biological Technology). The PCNA labeling index was expressed as the ratio of cells that were positively stained for PCNA to all epithelial cells. The expressions of tight junction proteins were expressed as the average optical density (the ratio of integrated optical density to the area of tissue) in at least 5 areas. All areas were randomly selected for counting at less than 200-fold magnification and all data expressed as the relative values to those of control piglets.

### 2.10. Plasma Antioxidative Capacity

The determination of superoxide dismutase (SOD), malondialdehyde (MDA), glutathione S-transferase (GST), glutathione peroxidase (GSH-Px), and total antioxidant capacity (T-AOC) levels in plasma were measured by spectrophotometric methods according to manufacturer instructions of assay kits (Nanjing Jiancheng, Nanjing, China). In brief, SOD, GST, and GSH-Px were analyzed by the xanthine oxidase-xanthine reaction method and reduced glutathione method, respectively. T-AOC was detected by ferric-reducing/antioxidant power reaction method, and MDA capacity was assayed by the 2-thiobarbituric acid method. All samples were measured by a UV/visible spectrophotometer (UV-2450, Shimadzu, Kyoto, Japan).

### 2.11. Statistical Analysis

Data were analyzed by the analysis of variance, using the General Linear Models procedure of the SPSS 20.0 (SPSS Inc., Chicago, IL, USA). Significant differences between means were determined using Tukey's multiple comparison tests. Results on the column chart expressed as the mean ± standard error of mean. Results on the tables expressed as the mean and standard error of mean (SEM). Differences were declared as significant at *P* < 0.05, and trends toward significance are discussed at 0.05 ≤ *P* < 0.10.

## 3. Results

### 3.1. Growth Performance

Compared with the control, DON exposure reduced average daily gain (ADG) and gain to feed ratio (G/F) (Table 2). Orally administered RAPA led to poorer growth performance, further reducing the ADG and average daily feed intake (ADFI) and gain: feed ratio relative to the DON group. Conversely, CQ treatment alleviated the effects of DON with growth performance measures nearing those of the control group.

### 3.2. Beclin1, P62, LC3, and *β*-Actin Analysis

**Figure 1:**
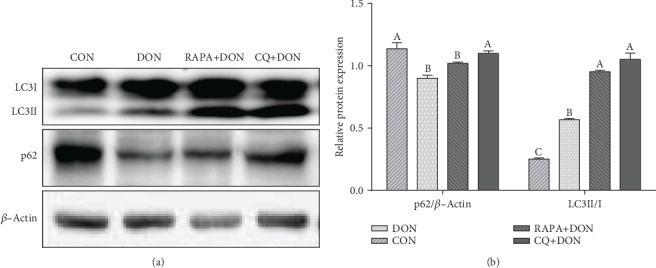
Autophagy validation in the jejunum of weaning piglets. Western blots results and abundance of p62 and LC3 protein abundance (the ratio of LC3IIand LC3I). Dietary treatment: CON: saline solution; DON: deoxynivalenol diet and saline solution; RAPA+DON: rapamycin and deoxynivalenol diet; CQ+DON: chloroquine and deoxynivalenol diet. Data are expressed as mean ± SEM (*n* = 6), four models chosen, ^a,b,c^Values within a row with different superscripts differ significantly (*P* < 0.05).

The group receiving DON and RAPA exhibited a higher ratio of LC3II/LC3I protein and a lower level of p62 relative to the control group, while CQ treatment increased the ratio of LC3II/LC3I and expression level of p62 (Figure 1). These findings suggest that DON and RAPA induced autophagy and CQ alleviated autophagy.

### 3.3. Cytokine Profiles

**Figure 2:**
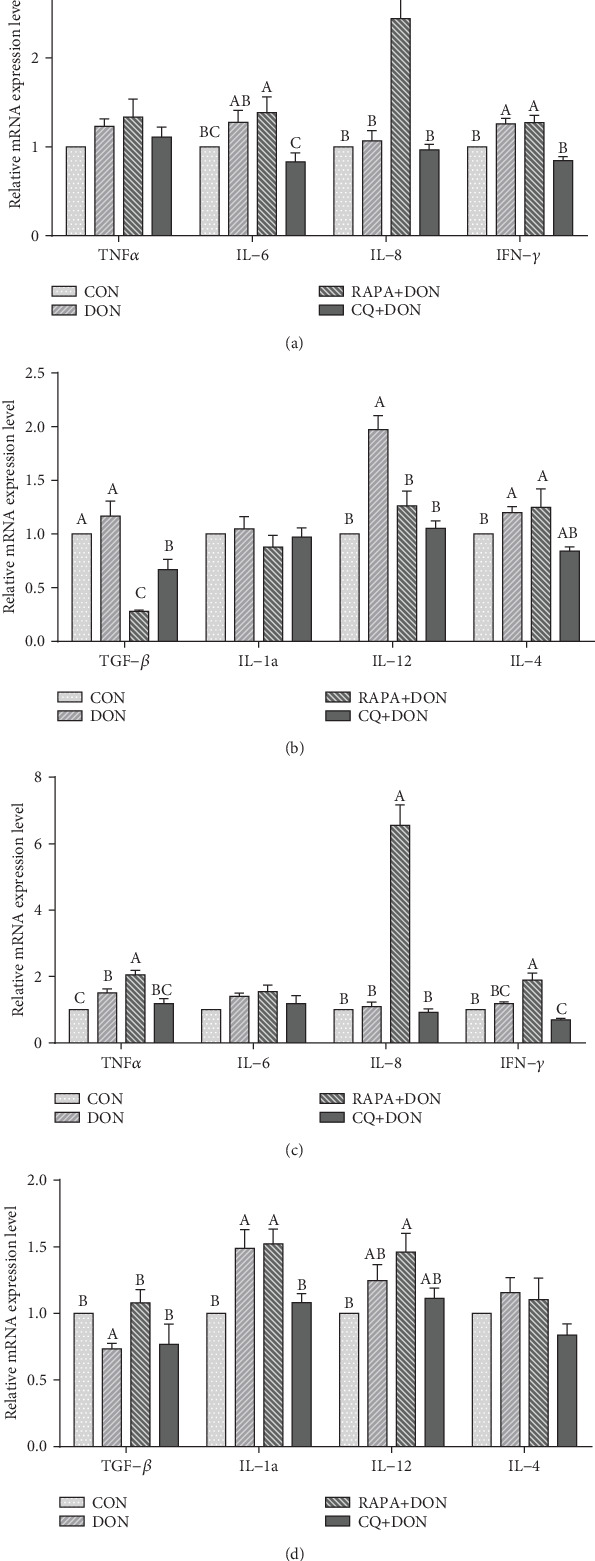
Relative mRNA levels of genes (TNF*α*, IL-6, IL-8, IFN-*γ*, TGF-*β*1, IL-1*α*, IL-12, IL-4) related to cytokine in the jejunum (a, b) and ileum (c, d) of weanling piglets. The real-time PCR method was employed, and *β*-actin was used as an internal control. Dietary treatment: CON: saline solution; DON: deoxynivalenol diet and saline solution; RAPA+DON: rapamycin and deoxynivalenol diet; CQ+DON: chloroquine and deoxynivalenol diet. Data are expressed as mean ± SEM (*n* = 6), four models chosen. ^a,b,c^Mean values with different letters were considered to be significantly different (*P* < 0.05). If “ab” is on top of the column the measurement is neither significant compared to “a” or “b”, the same of other.

Exposure to DON yielded increased serum concentrations of IL-6, IL-12, IL-1*β*, TNF-*α*, IL-10, and IFN-*γ*, but decreased TGF-*β* content (Table 3). Consistent with this, we found greater expression of IL-6, IL-12, IL-1*β*, and TNF-*α* mRNA in the jejunum (Figure 2), as well as a lower level of TGF-*β* mRNA. Relative to the DON-only group, the DON + RAPA group showed a greater increase in the serum and jejunum concentrations of IL-6, IL-8, and TNF-*α*, while CQ significantly decreased the levels of IL-6, IL-12, IL-1*β*, and TNF-*α* and increased the expression of factor TGF-*β* in both serum and jejunum to levels similar to the control group.

### 3.4. Serum D-Lactate and Diamine Oxidase

The contents of diamine oxidase (DAO) and D-lactic acid of the DON-treated group were significantly higher than those in the control group (Table 4). RAPA treatment further increased serum D-lactic acid concentration, while CQ treatment decreased the levels of both DAO and D-lactic acid in serum.

### 3.5. Intestinal Morphology

**Figure 3:**
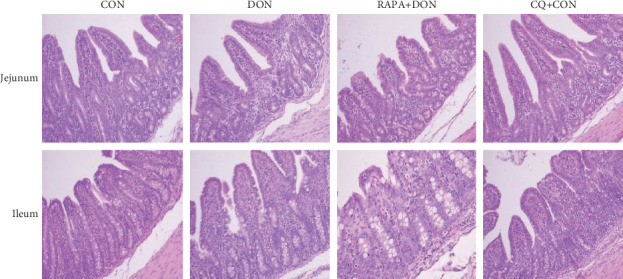
Morphology analysis representative picture (magnification ×100) in the jejunum and ileum of weanling piglets (*n* = 6). Dietary treatment: CON: saline solution; DON: deoxynivalenol diet and saline solution; RAPA+DON: rapamycin and deoxynivalenol diet; CQ+DON: chloroquine and deoxynivalenol diet.

The villus height of jejunum and ileum of DON-treated piglets was significantly lower than that of control piglets (Figure 3, Table 5). RAPA treatment further decreased villus height and increased the ratio of villus height to crypt depth (V/C), while CQ significantly increased villus height, indicating the amelioration of DON-induced injury to the jejunum and ileum villi.

### 3.6. E-Cadherin, Occludin, ZO-1, and Integrin

**Figure 4:**
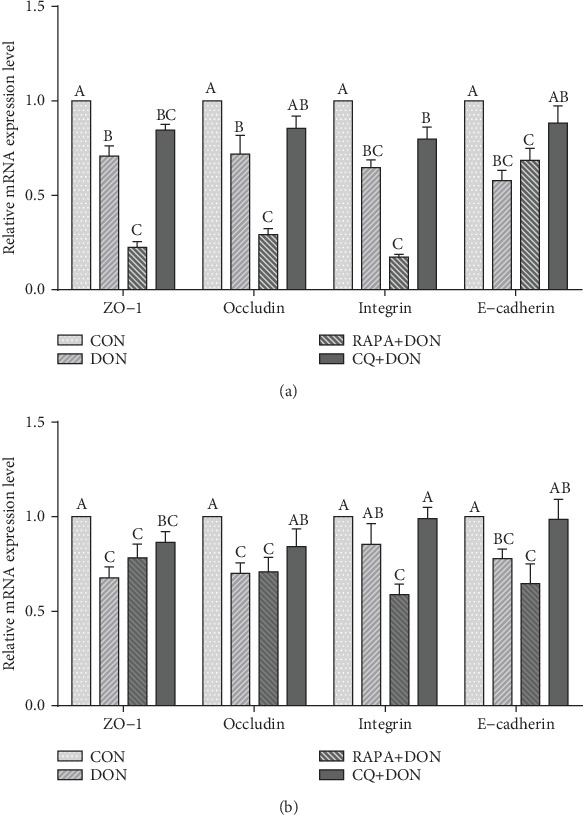
Relative mRNA levels of genes (ZO-1, occludin, integrin, and E-cadherin) related to tight junction and adherent junction in the jejunum (a) and ileum (b) of weanling piglets. The real-time PCR method was employed, and *β*-actin was used as an internal control. Dietary treatment: CON: saline solution; DON: deoxynivalenol diet and saline solution; RAPA+DON: rapamycin and deoxynivalenol diet; CQ+DON: chloroquine and deoxynivalenol diet. Data are expressed as mean ± SEM (*n* = 6), four models chosen. ^a,b,c^Mean values with different letters were considered to be significantly different (*P* < 0.05). If “ab” is on top of the column the measurement is neither significant compared to “a” or “b”, the same of other. Differences were assessed within each target gene.

DON treatment decreased the mRNA expression of E-cadherin, occludin, zonula occludens-1 (ZO-1), and integrin in the jejunal and ileal mucosa relative to controls (Figure 4). RAPA further downregulated the expression of E-cadherin, occludin, ZO-1, and integrin mRNA in the jejunum, while CQ treatment ameliorated the expression of tight junction protein. Under DON treatment, integrin mRNA expression in the ileal mucosa was lower than in controls. There were no differences in the expression of any investigated genes between DON and CQ treatments.

**Figure 5:**
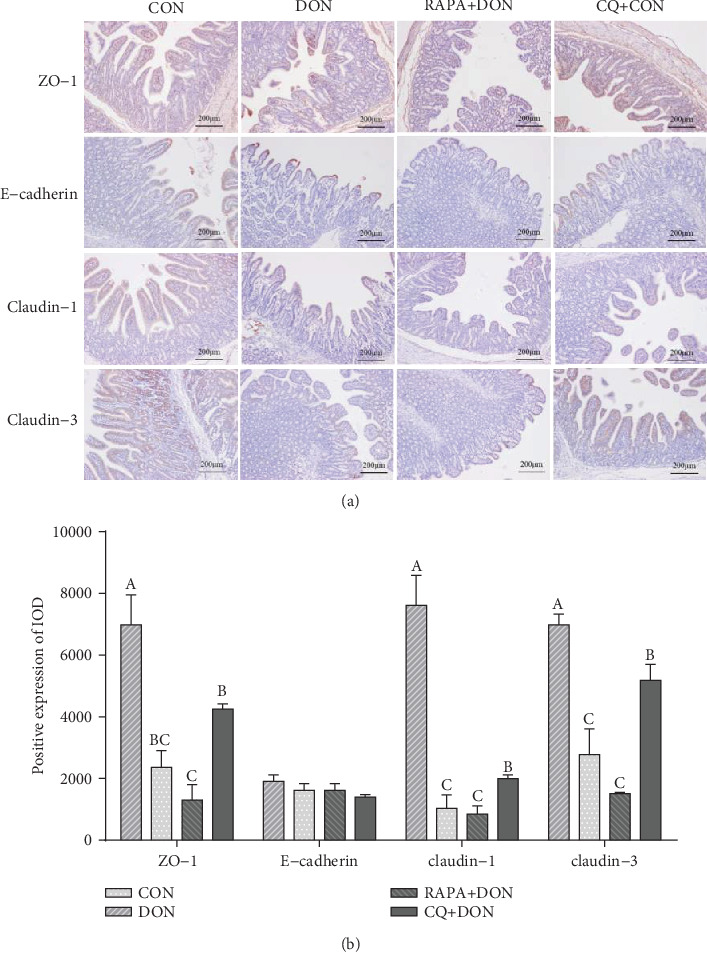
Immunohistochemical staining representative picture (magnification ×100) in jejunal mucosa (a). Relative positive expression of the proteins (ZO-1, E-cadherin, claudin-1, and claudin-3) related to tight junction and adherent junction in the jejunum of weanling piglets (b). Dietary treatment: CON: saline solution; DON: deoxynivalenol diet and saline solution; RAPA+DON: rapamycin and deoxynivalenol diet; CQ+DON: chloroquine and deoxynivalenol diet. Data are expressed as mean ± SEM (*n* = 6), four models chosen. ^a,b,c^Mean values with different letters were considered to be significantly different (*P* < 0.05). If “ab” is on top of the column the measurement is neither significant compared to “a” or “b”, the same of other.

The expression of E-cadherin, ZO-1, claudin-1, and claudin-3 in the jejunum of DON-treated were obviously lower than in the control group. In comparison, CQ treatment increased the optical density of tight junction-associated proteins and upregulated their expression (Figure 5).

### 3.7. Immunohistochemical Staining of Jejunal Mucosa PCNA

**Figure 6:**
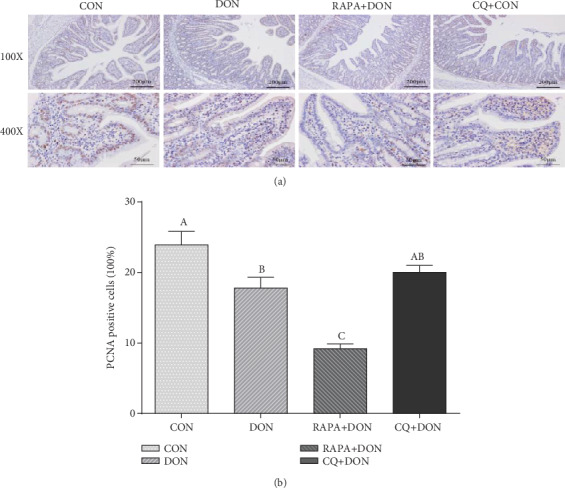
PCNA immunohistochemical staining representative picture (magnification ×100, and ×400) in jejunal mucosa (a), Positive expression of PCNA in the jejunum of weanling piglets (b). Dietary treatment: CON: saline solution; DON: deoxynivalenol diet and saline solution; RAPA+DON: rapamycin and deoxynivalenol diet; CQ+DON: chloroquine and deoxynivalenol diet. Data are expressed as mean ± SEM (*n* = 6), four models chosen. ^a,b,c^Mean values with different letters were considered to be significantly different (*P* < 0.05).

A representative image and labeling index of proliferating cell nuclear antigen (PCNA) immunohistochemical staining are shown in Figure 6. The percentage of PCNA-positive cells in the jejunum of piglets treated with DON was significantly lower than in the control group. In comparison, there was a significant decrease in the percentage of PCNA-positive cells in the RAPA group, while CQ treatment increased the PCNA labeling index.

### 3.8. Antioxidant Factors Profiles

**Figure 7:**
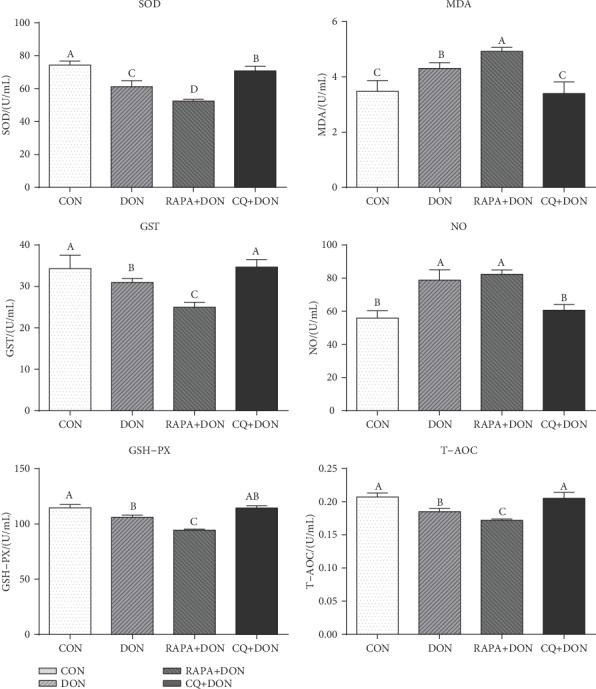
Antioxidant factors profiles in plasma. Dietary treatment: CON: saline solution; DON: deoxynivalenol diet and saline solution; RAPA+DON: rapamycin and deoxynivalenol diet; CQ+DON: chloroquine and deoxynivalenol diet. Data are expressed as mean ± SEM (*n* = 6), four models chosen. ^a,b,c^Mean values with different letters were considered to be significantly different (*P* < 0.05).

As shown in Figure 7, DON specimens exhibited lower levels of antioxidants, with remarkably downregulated concentrations of superoxide dismutase (SOD), glutathione S-transferase (GST), glutathione peroxidase (GSH-Px), and total antioxidant capacity (T-AOC), as well as upregulation of malondialdehyde (MDA). RAPA-treated group downregulation antioxidant ability, while the CQ-treated group exhibited improved antioxidant ability with higher concentrations of GSH-Px and GST relative to the DON group.

## 4. Discussion

DON causes toxicity such as growth retardation, weight loss, anorexia, and metabolic disorders in weanling piglets [26, 27]. In our study, DON led to a significant decrease in growth performance and average daily gain following the exposure to DON for 7 days. To explore the role of autophagy in conferring with DON *in vivo*, two reagents, autophagy inducer RAPA and autophagy inhibitor CQ, were used in the piglet as an animal model. The RAPA-treated group showed aggravated intestinal autophagy leading to further growth inhibition, while CQ alleviated DON damage with intestinal health indices comparable to the level of the control group, yielding improved growth performance with lower levels of autophagy.

The exacerbation of inflammatory responses contributes to increased intestinal damage. Autophagy is believed to play an important role in mediating inflammatory responses through clearing damaged organelles and misfolded proteins and reducing the levels of inflammatory cytokines or increasing levels of anti-inflammatory cytokines [28]. We found that treatment with RAPA yielded higher levels of proinflammatory cytokines in both blood and intestinal cells, triggering a severe inflammatory response with exacerbation of autophagy through RAPA and DON stimulation. In contrast, treatment with CQ yielded significantly lower inflammatory cytokines in blood and intestinal cells compared to a healthy control group, as well as reduced autophagy. These results indicated that, in piglets exposed to DON, RAPA led to excessive autophagy, more proinflammatory cytokines, and severe inflammatory response, while CQ downregulated proinflammatory factors and autophagy, enhancing growth performance.

Intestinal villus height, crypt depth, and tight junction proteins, and indices of cell production, maturation, and secretory function were further negatively affected by treatment with RAPA in DON-exposed piglets. Meanwhile, in keeping with other indices of comparison, CQ-treated piglets showed ameliorated intestinal mucosal morphology. Although a normal physiological rate of autophagy plays a role in cytoprotective mechanisms in response to toxin stress [15, 29–31], our results suggest that excessive or uncontrolled autophagy due to RAPA + DON induces physiological dysfunction and severe inflammatory reactions, leading to intestinal damage and decreased growth performance. Piglets exposed to CQ showed inhibited lysosomal acidification, preventing the development of autophagy, and also showed levels of autophagy, inflammatory cytokines, and intestinal mucous barrier features comparable to a DON-free group. This suggests that moderate alteration of autophagy eases the inflammatory response and yields concomitant enhancement of growth performance with a better intestinal barrier function. In addition to regulating autophagy, CQ can ameliorate cellular function by targeting a variety of proteases to suppress their activity, inhibiting protein hydrolysis, glycolipid generation, and other metabolic pathways [17], which may serve a protective role during toxin stress.

Oxidative stress is another known mechanism by which DON causes damage to the body, producing a large number of free radicals [32, 33] and consuming antioxidants in the body. At the same time, DON inhibits the activity of antioxidant enzymes such as SOD and catalase, resulting in excessive production of oxidation markers such as lipid peroxidation and MDA. These substances can cause severe damage to the structure of the cell membrane, destroy the antioxidant balance, and cause cell dysfunction [34]. This study found that DON reduced the activity of SOD and GSH in plasma, and CQ treatment restores the activity of SOD and GSH in plasma and reduced the content of MDA relative to the DON group. This suggests that the CQ-treated group increases the level of antioxidant enzymes in the blood that in turn accelerated neutralize ROS and play an antioxidant role [35]. In addition, CQ treatment increased the content of nitric oxide (NO), indicating the regulation balance between oxidation and antioxidant system *in vivo* [35], which an appropriate amount of NO can remove oxygen free radicals, and thus, protecting the cell membrane and reducing tissue damage [36].

## 5. Conclusion

Our results demonstrated that CQ significantly improves intestinal barrier function, inflammation response, and antioxidant ability *in vivo*. It may, therefore, be useful as a potential feed additive agent against mycotoxicity in piglets.

## Figures and Tables

**Table 1:** Primers used for real-time quantitative PCR.

Genes	Accession no.	Primers	Sequences (5′-3′)
*β*-Actin	XM_003124280.3	Forward	GGATGCAGAAGGAGATCACG
		Reverse	ATCTGCTGGAAGGTGGACAG

GAPDH	NM_001206359.1	Forward	ATCCTGGGCTACACTGAGGAC
		Reverse	AAGTGGTCGTTGAGGGCAATG

E-cadherin	NM_001163060.1	Forward	GAAGGAGGTGGAGAAGAGGAC
		Reverse	AGAGTCATAAGGTGGGGCAGT

Occludin	NM_001163647.2	Forward	AGAGTCATAAGGTGGGGCAGT
		Reverse	CGCCCGTCGTGTAGTCTGTC

ZO-1	XM_005659811.1	Forward	TACCCTGCGGCTGGAAGA
		Reverse	GGACGGGACCTGCTCATAACT

Integrin	NM_213968.1	Forward	GCAGTTTCAAGGTCAAGATGG
		Reverse	AGCAGGAGGAAGATGAGCAG

TNF*α*	NM_214022.1	Forward	ACAGGCCAGCTCCCTCTTAT
		Reverse	CCTCGCCCTCCTGAATAAAT

IL-6	NM_214399.1	Forward	TCCAGCATCATTGCATCATC
		Reverse	GGCTCCACTCACTCCACAAG

IL-8	NM_213948.1	Forward	TGAGAAGCAACAACAACAGCA
		Reverse	CAGCACAGGAATGAGGCATA

IFN-*γ*	NM_213948.1	Forward	TTCAGCTTTGCGTGACTTTG
		Reverse	GGTCCACCATTAGGTACATCTG

TGF-*β*1	NM_001173023.1	Forward	AAGCGGCAACCAAATCTATG
		Reverse	CCCGAGAGAGCAATACAGGT

IL-1*α*	NM_214029.1	Forward	ACCCGACTGTTTGTGAGTGC
		Reverse	TTCCCAGAAGAAGAGGAGACTG

IL-12	NM_213993.1	Forward	ATCTCGGTTGGTGTTGTTCC
		Reverse	GGGTATCTCGTCCTCTGTCC

IL-4	NM_214123.1	Forward	CCCGAGTGTCAAGTGGCTTA
		Reverse	TGATGATGCCGAAATAGCAG

**Table 2:** Effect of dietary RAPA and CQ on growth performance following exposure to DON.

Parameters	CON	DON	RAPA+DON	CQ+DON	SEM	*P* value
ADG (g/d)	182.60^a^	136.52^b^	-23.80^c^	171.29^ab^	9.37	<0.01
ADFI (g/d)	255.69^a^	248.67^a^	116.87^b^	232.86^a^	8.16	<0.01
G/F	0.72^a^	0.54^b^	-0.22^c^	0.72^a^	0.06	<0.01

CON: saline solution; DON: deoxynivalenol diet and saline solution; RAPA+DON: rapamycin and deoxynivalenol diet; CQ+DON: chloroquine and deoxynivalenol diet. ^a,b,c^Values within a row with different superscripts differ significantly (*P* < 0.05).

**Table 3:** Effect of dietary RAPA and CQ on serum inflammatory cytokine level following exposure to DON.

Parameters	CON	DON	RAPA+DON	CQ+DON	SEM	*P* value
IL-6 (pg/mL)	553.07^c^	652.04^b^	718.29^a^	592.32^c^	15.93	<0.01
IL-8 (pg/mL)	34.32^b^	33.42^b^	43.88^a^	31.76^b^	1.31	<0.01
IL-12 (pg/mL)	36.03^c^	46.10^ab^	52.35^a^	37.85^bc^	1.89	<0.01
IL-1*β* (pg/mL)	366.27^ab^	431.12^a^	381.23^ab^	314.97^b^	13.78	0.02
TNF-*α* (pg/mL)	102.34^b^	124.08^a^	131.36^a^	111.92^b^	4.54	0.05
TGF-*β* (pg/mL)	441.70^b^	336.34^c^	632.91^a^	421.70^b^	28.73	<0.01
IgG (g/L)	1.47	1.63	1.47	1.56	0.05	0.37
IgM (g/L)	0.43^a^	0.53^a^	0.14^b^	0.48^a^	0.03	< 0.01

CON: saline solution; DON: deoxynivalenol diet and saline solution; RAPA+DON: rapamycin and deoxynivalenol diet; CQ+DON: chloroquine and deoxynivalenol diet. ^a,b,c^Values within a row with different superscripts differ significantly (*P* < 0.05). If “ab” is in the marked place the measurement is neither significant compared to “a” or “b”, the same as other.

**Table 4:** Effect of dietary RAPA and CQ on Serum Diamine Oxidase and D-lactate following exposure to DON.

Parameters	CON	DON	RAPA+DON	CQ+DON	SEM	*P* value
DAO (mmol/L)	1.40^b^	1.70^a^	0.60^c^	1.28^b^	0.09	<0.01
D-lactose (*μ*g/mL)	76.093^c^	86.76^b^	95.77^a^	76.07^c^	2.69	<0.01

CON: saline solution; DON: deoxynivalenol diet and saline solution; RAPA+DON: rapamycin and deoxynivalenol diet; CQ+DON: chloroquine and deoxynivalenol diet. ^a,b,c^Values within a row with different superscripts differ significantly (*P* < 0.05). If “ab” is in the marked place the measurement is neither significant compared to “a” or “b”, the same of other.

**Table 5:** Effect of dietary RAPA and CQ on intestinal morphology following exposure to DON.

Parameters	CON	DON	RAPA+DON	CQ+DON	SEM	*P* value
Jejunum						
Villus height (*μ*m)	352.63^a^	323.54^b^	259.51^c^	341.10^a^	19.93	<0.01
Crypt depth (*μ*m)	86.70^b^	97.31^a^	78.21^c^	90.20^b^	3.46	<0.01
Villus height: crypt	4.07^a^	3.33^c^	3.32^c^	3.79^b^	0.09	<0.01
Wall thickness (*μ*m)	159.24^bc^	182.40^a^	150.12^c^	166.48^b^	4.72	<0.01
Goblet cell number	8.50	9.17	8.17	7.67	0.14	0.32
Lymphocyte number	31.50^a^	30.00^a^	27.83^b^	30.67^a^	0.10	<0.01
Ileum						
Villus height (*μ*m)	325.30^a^	267.71^c^	223.00^d^	297.78^b^	20.14	<0.01
Crypt depth (*μ*m)	89.19^a^	88.27^a^	77.80^b^	87.54^a^	2.81	<0.01
Villus height: crypt	3.65^a^	3.16^b^	2.87^c^	3.42^a^	0.14	<0.01
Wall thickness (*μ*m)	160.20^a^	168.36^a^	94.80 ^b^	159.06^a^	17.59	<0.01
Goblet cell number	10.17	9.33	10.67	11.67	0.29	0.29
Lymphocyte number	31.50	29.67	30.50	29.33	0.10	0.25

CON: saline solution; DON: deoxynivalenol diet and saline solution; RAPA+DON: rapamycin and deoxynivalenol diet; CQ+DON: chloroquine and deoxynivalenol diet. ^a,b,c^Values within a row with different superscripts differ significantly (*P* < 0.05). If “ab” is in the marked place the measurement is neither significant compared to “a” or “b”, the same of other.

## Data Availability

All data during the study are available from the corresponding author by request.

## Abbreviations

mTOR: Mammalian target of rapamycin
LC3: The microtubule-associated protein 1 light chain 3
P62: P62/SQSTM1
IFN-*γ*: Interferon-*γ*
IL: Interleukin
TGF-*β*: Transforming growth factor-*β*
TNF-*α*: Tumor necrosis factor-*α*
RAPA: Rapamycin
CQ: Chloroquine
DON: Deoxynivalenol
IgG: Immunoglobulin G
IgM: immunoglobulin M
ZO-1: zonula occludens-1
G/F: gain: feed ratio.

## Additional Points

*Key Contribution.* These findings provide evidence of the roles of RAPA and CQ in DON-exposed piglets and indicated a link between autophagy and alteration of the inflammatory response as well as intestinal morphology *in vivo*.
